# Immunoexpression of cleaved caspase-3 shows lower apoptotic area indices in lip carcinomas than in intraoral cancer

**DOI:** 10.1590/1678-775720160156

**Published:** 2016

**Authors:** Ana Flávia Schueler de Assumpção LEITE, Vagner Gonçalves BERNARDO, Luisa Aguirre BUEXM, Eliene Carvalho da FONSECA, Licínio Esmeraldo da SILVA, Danielle Resende Camisasca BARROSO, Simone de Queiroz Chaves LOURENÇO

**Affiliations:** 1 - Universidade Federal Fluminense, Faculdade de Odontologia, Niterói, RJ, Brasil.; 2 - Universidade do Estado do Rio de Janeiro, Instituto de Biologia Roberto de Alcântara Gomes, Departamento de Bioquímica, Rio de Janeiro, RJ, Brasil.; 3 - Instituto Nacional de Câncer José Alencar Gomes da Silva, Centro de Pesquisas, Programa de Carcinogênese Molecular, Rio de Janeiro, RJ, Brasil.; 4 - Universidade Federal Fluminense, Faculdade de Medicina, Departamento de Patologia, Niterói, RJ, Brasil.; 5 - Universidade Federal Fluminense, Instituto de Matemática, Departamento de Estatística, Niterói, RJ, Brasil.; 6 - Universidade Federal Fluminense, Faculdade de Odontologia, Departamento de Formação Específica, Nova Friburgo, RJ, Brasil.; 7 - Universidade Federal do Espírito Santo, Faculdade de Odontologia, Departamento de Clínica-odontológica, Vitória, ES, Brasil.; 8 - Universidade Federal Fluminense, Faculdade de Odontologia, Programa de Pós-graduação em Odontologia, Niterói, RJ, Brasil.

**Keywords:** Mouth neoplasms, Carcinoma, Leukoplakia, Cheilitis, Apoptosis, Caspase-3

## Abstract

**Objective:**

This study aimed to evaluate apoptosis by assessing cleaved caspase-3 immunoexpression in hyperplastic, potentially malignant disorder (PMD), and malignant tumors in intraoral and lower lip sites.

**Material and Methods:**

A retrospective study using paraffin blocks with tissues from patients with inflammatory fibrous hyperplasia (IFH), actinic cheilitis, oral leukoplakia, lower lip and intraoral squamous cell carcinoma (SCC) was performed. The tissues were evaluated by immunohistochemical analysis with anti-cleaved caspase-3 antibody. Apoptotic area index was then correlated with lesion type.

**Results:**

From 120 lesions assessed, 55 (46%) were cleaved caspase-3-positive. The SCC samples (n=40) had the highest apoptotic area indices (n=35; 87.5%). Significant differences were detected between SCCs and PMDs (p=0.0003), as well as SCCs and IFHs (p=0.001), regarding caspase-3 immunopositivity. Carcinomas of the lower lip had lower apoptotic area indices than intraoral cancer (p=0.0015).

**Conclusions:**

Cleaved caspase-3 immunoexpression showed differences in oral SCCs and PMDs and demonstrated a distinct role of apoptosis in carcinogenesis of intraoral and lower lip cancer. In future, the expression of cleaved caspase-3 with other target molecules in oral cancer may be helpful in delineating the prognosis and treatment of these tumors.

## INTRODUCTION

Oral cancer is the 11^th^ most common cancer in the world^6^. In Brazil, data from the Brazilian National Cancer Institute indicate that oral cancer is the fifth most common cancer among males and the twelfth among females, with a total of 15,490 new cases expected in 2016. More than 90% of oral cancer cases are squamous cell carcinoma (SCC)^12^. Early detection of high-risk premalignancy can decrease morbidity and mortality associated with oral cancer^22^. Oral leukoplakia (OL) and actinic cheilitis (AC) are premalignant lesions that show histological diversity and are associated with an overall increased risk for the development of invasive oral SCC^7,22,30^.

Risk factors for the development of intraoral and extraoral lesions are distinct. Strong associations between intraoral SCC and alcohol consumption and tobacco smoking are well established. In contrast, cancers of the lip vermilion are strongly associated with chronic sun exposure, although they are also related to the cigarette placement site on the lip^2,17,19,29^.

Dysregulation of apoptotic pathways is one of the fundamental processes in carcinogenesis. Apoptosis is regulated by several proteins that can inhibit (e.g., Bcl-2, Bcl-x, mutant p53, survivin) or promote (e.g., Bax, caspase) cell death^5,23,26^.

Caspases are a family of cysteine proteases involved in signaling and execution of apoptotic cell death. They are synthesized as pro-enzymes and activated by proteolytic cleavage, and may cleave other caspases as part of the apoptotic signaling cascade. Caspase-3 is a major effector caspase. Its proenzyme is expressed in a wide range of tissues, including those with a high tissue turnover, such as intestinal epithelium and epidermis. Active caspase-3 staining was considered a reliable method of apoptotic index scoring^8^.

Investigations of apoptotic cells levels in oral SCC are contradictory. Some studies showing elevated^8,16,18^ expression for oral SCC when compared with normal mucosa and others have suggested that apoptosis decreases as histological abnormality increases^24^.

Considering the different etiopathogeneses involved in the potentially malignant disorder (PMD) and SCC of intraoral and lower lip lesions, we hypothesized that a distinct apoptotic index was associated with these pathologies. Therefore, the purpose of this study was to characterize and compare cleaved caspase-3 immunoexpression in hyperplastic inflammatory lesions, PMDs, and malignant tumors.

## MATERIAL AND METHODS

### Study population

Formalin-fixed, paraffin-embedded tissue blocks with biopsy specimens were analyzed (n=120; 20 OL cases, 16 with and 4 without dysplasia; 20 AC cases, 15 with and 5 without dysplasia; 40 oral SCC cases: 20 intraoral SCCs and 20 lower lip SCCs; 40 inflammatory fibrous hyperplasia (IFH) cases: 20 intraoral IFH and 20 lower lip IFH). These blocks were retrieved from the files of the Department of Pathology at Antonio Pedro University Hospital (Federal Fluminense University). Hematoxylin-eosin-stained slides were reviewed, and initial diagnoses reconfirmed. This study was approved by the institutional ethics committee (CMM/HUAP 33/06).

### Immunohistochemistry

Immunohistochemistry was performed on serial paraffin 4-µm sections of each block mounted on silane-pretreated glass slides (Sigma Chemicals, St. Louis, MO, USA), using a streptavidin-biotin peroxidase technique. Deparaffinized sections were immersed in 3% hydrogen peroxide for 30 min to block endogenous peroxidase activity and heated (94-96°C) for 30 min in 1 mM citric acid buffer (pH 6.0) for antigen retrieval (Target antigen retrieval solution, DAKOCytomation, Carpinteria, CA, USA). Sections from each block were incubated in 1:600 diluted solution of rabbit polyclonal antibody to cleaved caspase-3 protein, for 16-18 h at 4°C, as primary antibody (*Asp 175,* Cell Signaling Technology, Danvers, MA, USA). Peroxidase staining was visualized with 3,3′-diaminobenzidine (DAB^+^ Liquid DAB substrate, Chromogen system*,* DAKOCytomation, Carpinteria, CA, USA). Sections were counterstained with Harris’ hematoxylin, dehydrated, cleared in xylene, and mounted. The primary antibody was omitted and replaced by an antibody diluent (Antibody diluent with background reducing components solution, DAKOCytomation, Carpinteria, CA, USA) for negative control, resulting in immunonegative slides in all instances. Positive controls comprised oral lichen planus sections, since several apoptotic keratinocytes are usually present in this lesion.

### Quantitative Immunohistochemical Analysis

A binary classification (positive vs negative) was used to score the immunohistochemistry in the distinct lesions and sites evaluated. The positive slides were quantitatively evaluated by apoptotic area index.

Digital image analysis was used for quantification of immunostaining by Nikon Microscope Eclipse E400 (Nikon, Tokyo, Tokyo, Japan) and Evolution^TM^ MP Color 5.0 Mega-pixel Camera Kit (Media Cybernetics, Silver Spring, MD, EUA). Five fields (20×/0.4 Plan Achromat; objective lens; total area: 705.630 µm^2^), selected from hot-spot areas, were acquired per slide. Image analysis was performed with Image-Pro Plus 4.5 software (Media Cybernetics, Silver Spring, MD, USA).

United discrimination plane was used to segment the images (24-bit). This method, developed by one of the authors (VB)^3^ uses areas of specific staining from different images to determine the positivity discrimination plane, minimizing a possible visual variation in detection of immunostained areas over time when an interactive discrimination plane is used. To measure the total area, the discrimination plane was set at 0-255 in all RGB channels. Because immunohistochemical signals from epithelial tissue had to be separated from stromal signals, all lesion areas were manually outlined in all microscopic fields to increase counting specificity. Cytoplasmic positivity was expressed as an area index (positive area/total area) instead of a labeling index because it was not possible to distinguish whether a positive area corresponded to a specific cell or to an adjacent cell.

### Statistical methods

Shapiro-Wilk test was used to assess data normality. Kruskal-Wallis nonparametric test (H) and Mann-Whitney test (U) were used to compare apoptotic area indices within each group (intraoral - IFH, OL, and SCC; lower lip - IFH, AC, and SCC) and between groups. Binomial test and Dixon Q test were used in PMD quantitative result assessments. In all analyses, p<0.05 was considered significant.

## RESULTS

**Figure 1:**
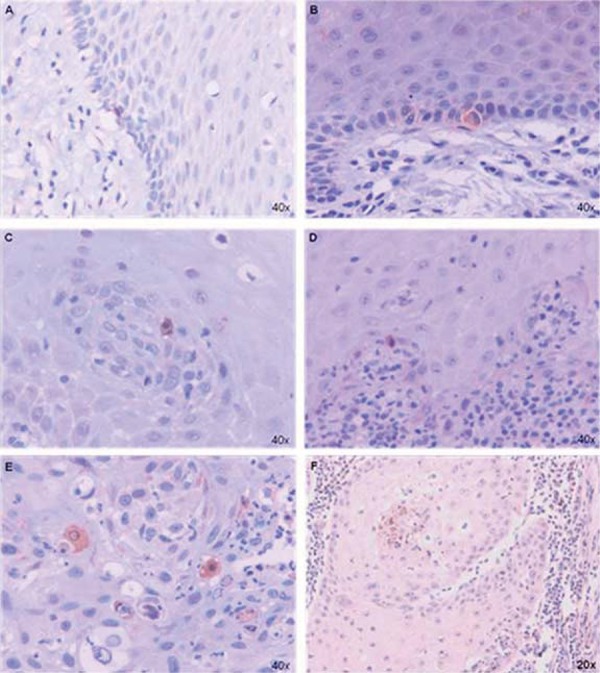
Immunohistochemical expression of cleaved caspase-3 in lesions. A- Intraoral oral leukoplasia (IFH). B- Lower lip IFH. C- oral leukoplasia (OL). D- actinic cheilitis (AC). E- Intraoral squamous cell carcinoma (SCC). F- Lower lip SCC

From 120 lesions assessed, 55 (46%) stained positive with anti-cleaved caspase-3 antibody (Table 1 and Figure 1). Cleaved caspase-3 immunoexpression for IFH was negative in most cases (intraoral IFH n=16/80%; lower lip IFH n=17/85%). In PMDs, OL with and without dysplasia also presented a higher number of negative cases (OL with dysplasia n=10/62.5%; OL without dysplasia n=4/100%). In the AC there was difference in immunoexpression between cases with and without dysplasia, AC with dysplasia had more negative cases (n=9/60%) and AC without dysplasia showed more positive cases (n=3/60%). In the SCC group, a higher frequency of positive cases (intraoral SCC n=20/100%; lower lip SCC n=15/75%) was observed.

**Table 1:** Immunohistochemical expression of cleaved caspase-3 in intraoral and lip IFH, OL, AC, and intraoral and lip SCC (n=120)

Lesion/Location	Immunohistochemical Expression	No. Cases	% Cases	Apoptotic Area Index (Average)
Intraoral IFH	Positive	4	20%	0.00011
	Negative	16	80%	
	Total	20	100%	
Lip IFH	Positive	3	15%	0.00007
	Negative	17	85%	
	Total	20	100%	
OL with dysplasia	Positive	6	37.5%	0.00045
	Negative	10	62.5%	
	Total	16	100%	
OL without dysplasia	Positive	0	0%	-
	Negative	4	100%	
	Total	4	100%	
AC with dysplasia	Positive	6	40%	0.00010
	Negative	9	60%	
	Total	15	100%	
AC without dysplasia	Positive	3	60%	0.00026
	Negative	2	40%	
	Total	5	100%	
Intraoral SCC	Positive	20	100%	0.00362
	Negative	0	0%	
	Total	20	100%	
Lip SCC	Positive	15	75%	0.00055
	Negative	5	25%	
	Total	20	100%	

IFH= inflammatory fibrous hyperplasia; OL= oral leukoplasia; AC= actinic cheilitis; SCC squamous cell carcinoma

**Figure 2:**
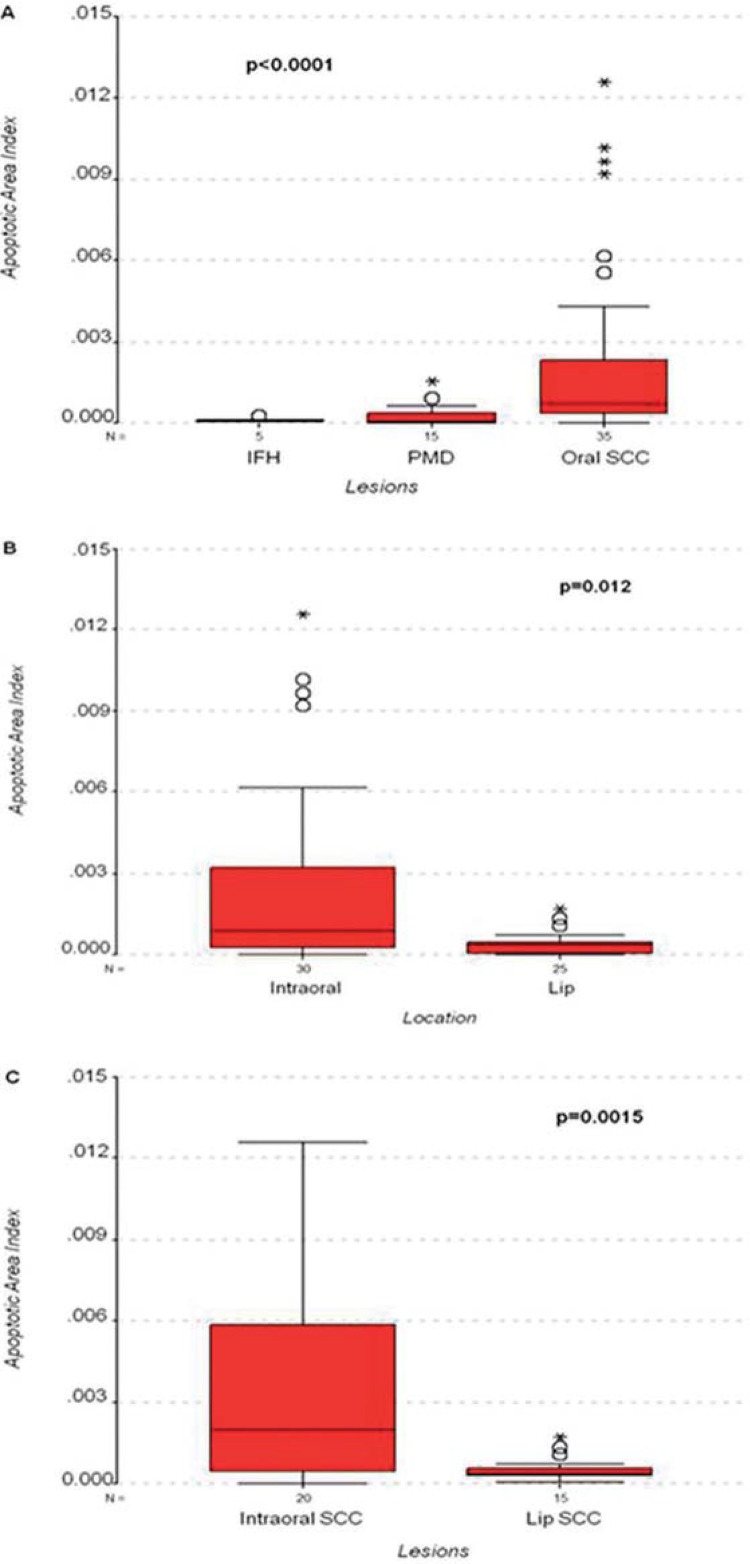
Distribution of area densities. A- inflammatory fibrous hyperplasia (IFH), potentially malignant disorder (PMD), and oral squamous cell carcinoma (SCC) lesions. B- Intraoral and lower lip locations. C- Intraoral squamous cell carcinoma (SCC) and lower lip SCC

Results are shown as mean±standard error. Kruskal-Wallis test showed statistical differences between indices when all lesion groups, irrespective of their location, were compared (H=18.879; d.f.=2; p<0.0001) (Figure 2A). The SCC group (n=35) had the highest apoptotic area indices (0.00231±0.00056) and Mann-Whitney test indicated highly significant differences in the following group comparisons: SCC vs. PMD (U=90; p=0.0003), and SCC vs. IFH (U=12; p=0.001). Area indices for PMD (n=15) and IFH (n=5) were 0.00027±0.00011 and 0.00011±0.00004, respectively. No statistically significant differences were detected when comparing area indices of these lesions (U=31; p=0.612).

Apoptotic area indices in intraoral lesions (n=30; 0.0025±0.0006) were significantly higher (U=241; p=0.012) than in lower lip lesions (n=27; 0.0004±0.00008). (Figure 2B). Indices in intraoral SCCs (n=20; 0.0036±0.0009) were significantly higher (U=62; p=0.0015) than in lower lip SCCs (n=15; 0.0005±0.0001) (Figures 1E-F and 2C).

No significant differences (U=26; p=0.955) were detected comparing area indices of OL (n=6; 0.0005±0.0003) and AC (n=9; 0.0002±0.00008) (Figure 1C-D). Because only one case of lower lip IFH showed cleaved caspase-3-positivity (n=1; 0.00007), comparisons between lower lip and intraoral IFHs (n=4; 0.00012±0.00005) were not possible (Figure 1A-B).

When grouping lesions according to their location, Kruskal-Wallis test showed statistical differences between indices of the lower lip group lesions (IFH, AC, SCC; H=6.514; d.f.=2; p=0.039). When compared with AC (n=9), SCCs (n=15) had higher apoptotic area indices and Mann-Whitney test showed a statistically significant difference between these groups (U=28; p=0.018). It was not possible to compare lower lip SCC or AC with IFH. In lower lip group, the occurrence of only one positive case for IFH failed to bring evidence of possible differences between AC and SCC. In the intraoral group (IFH, OL, SCC) Kruskal-Wallis test indicated statistical differences between indices of these lesion groups (H=12.144; d.f.=2; p=0.002). SCCs (n=20) had higher apoptotic area indices than IFH and OL, and Mann-Whitney test showed statistically significant differences between SCC and OL (n=6; U=17; p=0.007), and between SCC and IFH (U=4; p=0.002). No statistical differences were detected between area indices of OL and IFH lesions (U=11; p=0.914).

In the PMD group, all intraoral leukoplakia lesions staining positive with anti-cleaved caspase-3 antibody were characterized by epithelial dysplasia. No significant differences (U=26; p=0,955) were noted when comparing area indices for intraoral leukoplakias with dysplasia (n=6) and AC with dysplasia (n=6; 0.0001±0.00007). The apoptotic area index of AC without epithelial dysplasia was higher than in AC epithelial dysplasia cases (U=1; p=0.036).

## DISCUSSION

Apoptosis is a key aspect in the pathogenesis of many diseases, e.g., cancer, and in the response of neoplastic cells to systemic therapy^9,25,28^. Several studies in this area often compared normal and/or dysplastic epithelium with carcinoma^13,20,24,27^. IFH epithelium was treated as a control in our study because of the difficulty in collecting samples of normal epithelium. Areas of apparently benign epithelium adjacent to the malignant tissue were used in some studies as a normal control group^1^. However, this approach also has limitations because of field cancerization^13^, which asserts the occurrence of early genetic changes in histologically normal cells surrounding malignant tumors.

In our study, oral SCC had a higher apoptotic area index than other types of lesions (p<0.05) and IFH epithelium had the lowest apoptotic area index. Hague, et al.^8^ (2004) observed that the proportion of active caspase-positive cells in oral SCCs was markedly higher than in normal oral epithelium, confirming that the apoptotic index increases during oral carcinogenesis. It has been also shown that in normal epithelium, cells positive for cleaved caspase-3 were rare. Our results concur with these findings. The current study showed no statistical difference between apoptotic area indices of premalignant and IFH groups, however, cleaved caspase-3-positive specimens predominated in premalignant lesions when compared with IFH. Similarly, other studies have reported a progressive increase of apoptotic cells, from normal epithelium state, through epithelial dysplasia, until oral SCC development^16,18^.

In contrast, some studies suggested that the number of apoptotic cells decreased in oral lesions, as a result of increased molecular abnormalities in epithelial cells and cancer^20,24^. Tanda, et al.^27^ (2000) found no significant differences between the number of apoptotic cells in OL compared with normal oral mucosa. Considering the differences in apoptosis detection methods, analyses modes, and study models used in the investigations, inter-study result comparisons are difficult^21^.

Our results showed differences between intraoral and lip SCCs. Cleaved caspase-3 levels were significantly higher in intraoral SCCs compared with lower lip SCCs. Lip SCCs have the best 5-year survival rates (over 90%) of all mouth SCCs, demonstrating their distinctive biological behavior^29^. Alterations in p53 expression suggest that this proteins are involved in lower lip carcinogenesis^15^. *TP53* mutation caused by UV irradiation decreases the number of apoptotic keratinocytes in skin cancer^14^. Keratinocytes with wild type *TP53* are usually eliminated by apoptosis, via p53, and keratinocytes with UV-induced *TP53* mutation should be more susceptible to the tumor-promoting effects of UV radiation. These cells should survive, with an increased risk of becoming malignant^14^. Thus, it is important to emphasize the differences in molecular carcinogenesis of oral and lip SCCs, identifying proteins that may be employed as prognostic markers.

Molecular markers and immunoregulatory events predicting the malignant potential of AC are not well established and remain to be elucidated^7^. According to our data, the apoptotic area index of AC without epithelial dysplasia was higher than in cases with epithelial dysplasia. This too can be explained by UV radiation-caused *TP53* mutation for cases without epithelial dysplasia, inhibiting the induction of apoptosis, as previously discussed. In contrast, all OL cases positive for cleaved caspase-3 protein showed epithelium dysplasia, probably suggesting a different molecular mechanism depending on etiological factors involved in premalignant lesion formation.

Several studies reported the potential use of activated caspase-3 as a biomarker to predict tumor responses to treatments^11^, in association with other prognostic variables (e.g., vascular invasion, lymph node metastasis, advanced clinical stage, and size of tumor)^4^, suggesting that cleaved caspase-3 could be used as a potential factor to predict tumor progression and poor prognosis in several cancer types^10^. In the current study, evaluation of cleaved caspase-3 levels in tissue biopsies showed differences in apoptotic area indices between pathologies and locations, which should be further verified in surgical specimens and in prospective studies.

## CONCLUSIONS

In summary, the present study showed differences in the immunoexpression of cleaved caspase-3 in oral SCCs and PMDs. In addition, it showed a distinct role of apoptosis in carcinogenesis of intraoral and lower lip cancer, which may be helpful, in the future, in delineating the prognosis and treatment of these tumors. Further studies, correlating the expression of cleaved caspase-3 with other target molecules in oral cancer, should lead to the establishment of an effective prognostic immunohistochemical biomarker panel for oral carcinogenesis.
